# Identification of TRAMs as sphingolipid-binding proteins using a photoactivatable and clickable short-chain ceramide analog

**DOI:** 10.1016/j.jbc.2021.101415

**Published:** 2021-11-16

**Authors:** Yaqin Deng, Lin You, Yong Lu, Sungwon Han, Jingcheng Wang, Nikitha Vicas, Chuo Chen, Jin Ye

**Affiliations:** 1 Department of Molecular Genetics, University of Texas Southwestern Medical Center, Dallas, Texas, USA; 2 Department of Biochemistry, University of Texas Southwestern Medical Center, Dallas, Texas, USA

**Keywords:** ceramide, click chemistry, ligand-binding protein, protein translocation, sphingolipid, ANKLE2, ankyrin repeat and LEM domain–containing protein 2, CCR5, C–C chemokine receptor 5, CREB3L1, cAMP response element–binding protein 3-like 1, DHCer, dihydroceramide, DMEM, Dulbecco's modified Eagle's medium, ER, endoplasmic reticulum, ESI, electrospray ionization, FB1, fumonisin B1, pac, photoactivatable and clickable, RAT, regulated alternative translocation, SILAC, stable isotope labeling by amino acids in cell culture, SPTLC1, serine palmitoyltransferase long-chain base subunit 1, TK, thymidine kinase, TLC, TRAM/LAG1/CLN8, TM4SF20, transmembrane 4 L6 subfamily member 20, TRAM2, translocating chain-associated membrane protein 2

## Abstract

Ceramide is a lipid molecule that regulates diverse physiological and pathological reactions in part through inverting the topology of certain transmembrane proteins. This topological inversion is achieved through regulated alternative translocation (RAT), which reverses the direction by which membrane proteins are translocated across the endoplasmic reticulum during translation. However, owing to technical challenges in studying protein–ceramide interaction, it remains unclear how ceramide levels are sensed in cells to trigger RAT. Here, we report the synthesis of pac-C_7_-Cer, a photoactivatable and clickable short-chain ceramide analog that can be used as a probe to study protein–ceramide interactions. We demonstrate that translocating chain-associated membrane protein 2 (TRAM2), a protein known to control RAT of transmembrane 4 L6 subfamily member 20, and TRAM1, a homolog of TRAM2, interacted with molecules derived from pac-C_7_-Cer. This interaction was competed by naturally existing long-chain ceramide molecules. We showed that binding of ceramide and its analogs to TRAM2 correlated with their ability to induce RAT of transmembrane 4 L6 subfamily member 20. In addition to probing ceramide–TRAM interactions, we provide evidence that pac-C_7_-cer could be used for proteome-wide identification of ceramide-binding proteins. Our study provides mechanistic insights into RAT by identifying TRAMs as potential ceramide-binding proteins and establishes pac-C_7_-Cer as a valuable tool for future study of ceramide–protein interactions.

In addition to being the structural components of membranes, lipids have been increasingly recognized as second messengers for various signal transduction reactions by interacting with their protein sensors. Ceramide is one such lipid that mediates various reactions (1) including cytotoxic and cytostatic effects of chemotherapeutic reagents (2). Among these reagents, doxorubicin blocks proliferation of cancer cells by stimulating proteolytic activation of cAMP response element–binding protein 3-like 1 (CREB3L1), a membrane-bound transcription factor (3, 4, 5). In the absence of doxorubicin, CREB3L1 exists as an inactive transmembrane precursor, as cleavage of the protein is inhibited by transmembrane 4 L6 subfamily member 20 (TM4SF20), a polytopic transmembrane protein (6). Doxorubicin induces production of ceramide (4), which in turn reverses the direction through which the first transmembrane helix of TM4SF20 is translocated across the Sec61 translocon in the endoplasmic reticulum (ER) through a process designated as regulated alternative translocation (RAT) (7). As a result, the topology of newly synthesized TM4SF20 produced under this circumstance (referred as TM4SF20(B)) is completely opposite to that generated in the absence of ceramide (referred as TM4SF20(A)) (7). This topological inversion turns TM4SF20 from an inhibitor to an activator for proteolysis of CREB3L1 (7), a reaction enabling translocation of the N-terminal domain of CREB3L1 from membranes to the nucleus, where it activates transcription of genes that inhibit cell proliferation (4, 8).

Sphingolipids are also involved in inhibition of macrophage chemotaxis mediated by chemokine receptors including C–C chemokine receptor 5 (CCR5), a G protein–coupled receptor (9, 10). Lipopolysaccharide increases production of dihydroceramide (DHCer) in macrophages, which in turn inverts the topology of newly synthesized CCR5 through RAT. Since CCR5 with the inverted topology no longer functions as a chemokine receptor, DHCer-induced topological inversion of CCR5 explains at least in part why endotoxin-tolerated macrophages are resistant to chemotaxis (10, 11).

These observations suggest that ceramide/DHCer-induced topological inversion of certain transmembrane proteins through RAT is responsible for at least some of the sphingolipid-mediated signal transduction. However, it remains unclear how the sphingolipids are sensed in cells in order to trigger RAT. We previously reported that translocation chain-associated membrane protein 2 (TRAM2), a homolog of TRAM1 that is known to be a Sec61 accessory protein (12, 13), is involved in RAT of TM4SF20 (7). Both TRAM proteins contain a TRAM/LAG1/CLN8 (TLC) homology domain that is also present in ceramide synthase, which is postulated to bind sphingolipids (14). However, a direct interaction between TRAM proteins and ceramide has never been reported. In the current study, we demonstrated the TRAM–ceramide interaction through a photoactivatable and clickable analog of short-chain ceramide we synthesized. Our study suggests that TRAMs may be the sphingolipid sensor that regulates translocation of certain transmembrane proteins.

## Results

**Figure 1:**
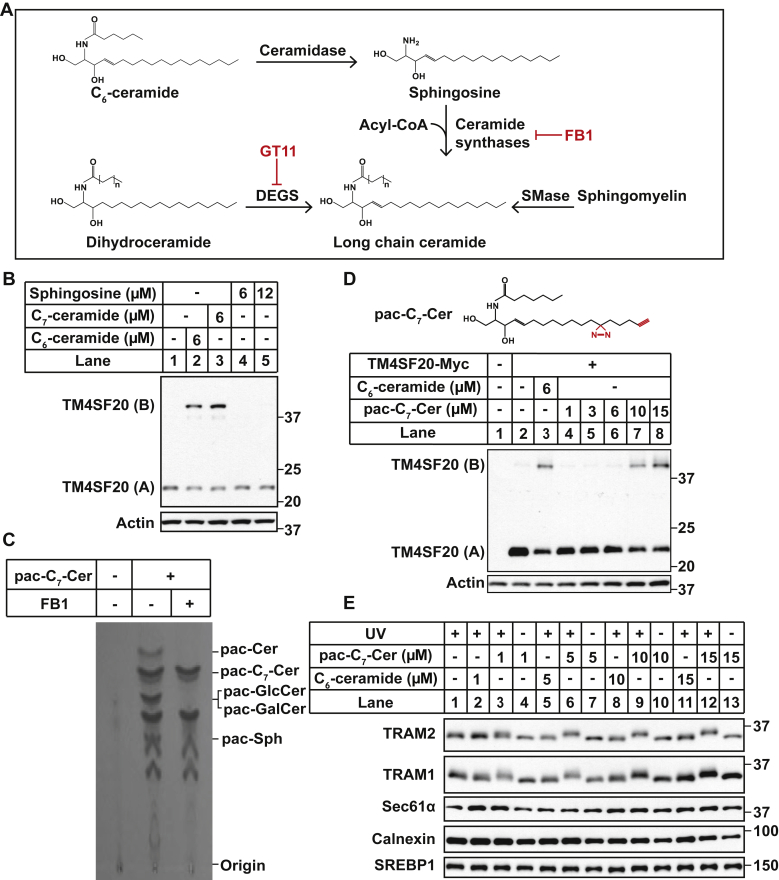
.**pac-C**_**7**_**-Cer is functional in inducing RAT of TM4SF20.***A*, ceramide metabolic pathways related to this study. *B*, A549/pTM4SF20 cells treated with indicated amounts of the compounds for 16 h were harvested for immunoblot analysis with antimyc to detect TM4SF20. *C*, lipids extracted from SV589 cells treated with 50 μM FB1 for 2 h followed by cotreatment with 4 μM pac-C_7_-Cer for 6 h as indicated were subjected to click reaction with 3-azido-7-hydroxycoumarin, separated by thin layer chromatography and analyzed by fluorescence imaging. The positions where indicated pac-sphingolipid markers were migrated on the same TLC plate were marked. *D*, SV589 cells transfected with pTK-TM4SF20-Myc or the empty vector (1 μg per well in 6-well plates) and treated with 6 μM C_6_-ceramide or indicated amounts of pac-C_7_-Cer for 16 h were harvested for immunoblot analysis with antimyc to detect TM4SF20. *D*, SV589 cells were treated with indicated amounts of the compounds for 1 h. Following UV irradiation for 15 min, cells were harvested for immunoblot analysis with the indicated antibodies. FB1, fumonisin B1; pac, photoactivatable and clickable; RAT, regulated alternative translocation; TLC, TRAM/LAG1/CLN8; TM4SF20, transmembrane 4 L6 subfamily member 20.

A major obstacle to study ceramide-mediated reactions is that ceramide is insoluble in ethanol or dimethyl sulfoxide, making it difficult to directly treat cells with the lipid. In a previous study, ceramide-binding proteins were identified by treating cells with a photoactivatable and clickable analog of sphingosine (pac-sphingosine), which is converted to ceramide in cells by a reaction catalyzed by ceramide synthases (Fig. 1*A*) (15, 16). However, pac-sphingosine was not applicable to identify the ceramide sensor that triggers RAT, as treating cells with sphingosine failed to induce RAT of TM4SF20 to produce TM4SF20(B), the topology of which is opposite to that of the protein produced in the absence of ceramide (TM4SF20(A)) (Fig. 1*B*, lanes 4 and 5). In contrast to sphingosine, treating cells with N-hexanoyl-d-erythro-sphingosine (C_6_-ceramide), a ceramide analog containing a shorter amide-linked acyl chain (Fig. 1*A*), induced RAT of TM4SF20 to produce TM4SF20(B) (Fig. 1*B*, lane 2) just as previously reported (7). The molecular weight of TM4SF20(B) is higher than that of TM4SF20(A) because the topologic inversion switches three potential N-linked glycosylation sites from cytosol to the lumen where they can be glycosylated (7). C_6_-ceramide is converted to naturally existing long-chain ceramide through two reactions catalyzed by ceramidase and ceramide synthases in cells (Fig. 1*A*) (17). If this conversion is required to stimulate RAT of TM4SF20, then the length of the artificial short acyl chain present in the ceramide analog should not be critical to induce this reaction, as it should be cleaved off and replaced by naturally existing long-chain fatty acids. Indeed, C_7_-ceramide was as effective as C_6_-ceramide in stimulating RAT of TM4SF20 (Fig. 1*B*, lane 3). Since short-chain ceramides trigger signaling reactions that cannot be induced by sphingosine, we decided to synthesize a photoactivatable and clickable analog of C_7_-ceramide (pac-C_7_-Cer) to identify ceramide-binding proteins. Using thin layer chromatography analysis, we determined that pac-C_7_-Cer was converted to long-chain pac-Cer and its glycosylation products in cells, and this conversion was inhibited by treatment with fumonisin B1 (FB1) (Fig. 1*C*), a ceramide synthase inhibitor that blocks conversion of short-chain ceramides to long-chain ceramides (18) (Fig. 1*A*).

Pac-C_7_-Cer (Fig. 1*D*) contains an ester-linked modified acyl chain including a photocrosslinkable diazirine group and a clickable alkynyl group. These modifications did not affect the signaling reactions mediated by ceramide, as treatment with the compound stimulated RAT of TM4SF20 (Fig. 1*D*). The first clue that pac-C_7_-Cer may interact with TRAM2 came from immunoblot analysis of cells treated with the compound. Treatment of cells with pac-C_7_-Cer followed by UV exposure increased the apparent molecular weight of TRAM2 in a concentration-dependent manner even without a click reaction that attached additional molecules to the compound (Fig. 1*E*, the *first panel*, lanes 3, 6, 9, and 12). This size shift did not occur if the cells were not subject to UV exposure, which is required to trigger crosslinking of the compound to its interacting proteins (Fig. 1*E*, the *first panel*, lanes 4, 7, 10, and 13). Nor did this shift occur when the cells were treated with C_6_-ceramide that is not crosslinkable (Fig. 1*E*, the *first panel*, lanes 2, 5, 8, and 11). This pattern of size shift was also observed for TRAM1 (Fig. 1*E*, the *second panel*). In contrast, other ER-localized transmembrane proteins, such as calnexin, Sec61α, and sterol regulatory element-binding protein 1, did not generate such a size shift in any one of the experimental conditions (Fig. 1*E*, *lower three panels*).

**Figure 2:**
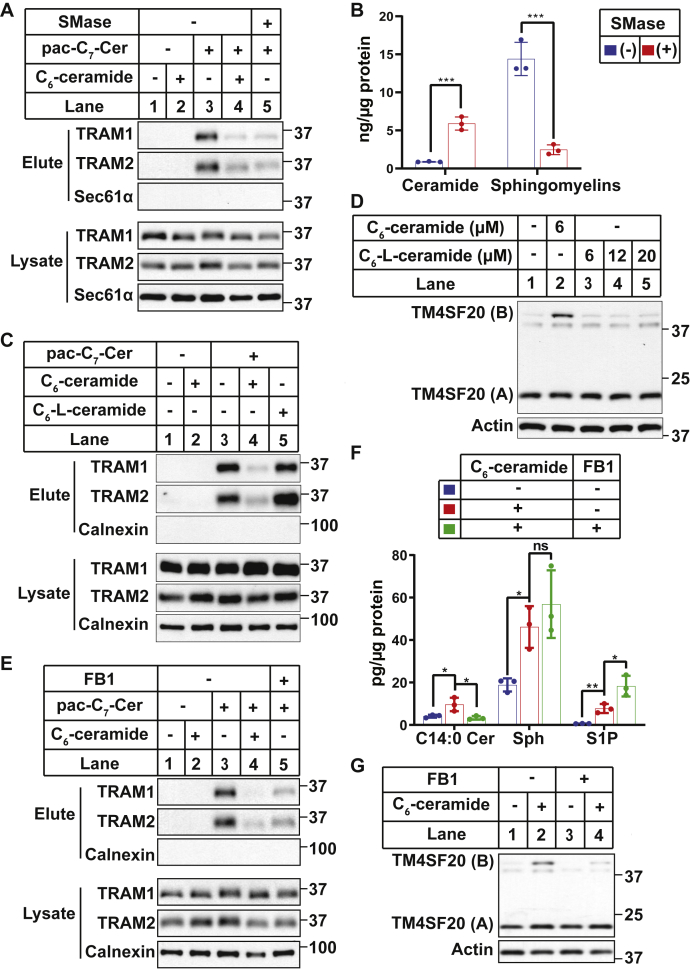
**Interaction of TRAM proteins with long-chain ceramide.***A*, SV589 cells were treated with 0.4 μM pac-C_7_-Cer, 0.4 μM (lane 2) or 80 μM (lane 4) C_6_-ceramide, or 0.4 U/ml sphingomyelinase (SMase). Cells were treated by these compounds for 1 h except for SMase, which included an additional pretreatment for 2 h. Following UV irradiation and click reactions attaching biotin, biotin-labeled proteins were isolated and subjected to immunoblot analysis with the indicated antibodies. *B*, the amounts of sphingomyelins and ceramide (summation of all species) in SV589 cells treated with or without 0.4 U/ml SMase for 3 h were determined by LC–MS. Results are reported as mean ± SD from triplicate incubation of a typical experiment. ∗∗∗*p* < 0.001 from two-tailed and unpaired *t* test. *C*, SV589 cells treated with 0.4 μM pac-C_7_-Cer and 80 μM C_6_-ceramide or C_6_-l-ceramide for 1 h were analyzed as described in (*A*). *D*, A549/pTM4SF20 cells treated with indicated amounts of the compounds for 6 h were harvested for immunoblot analysis with antimyc to detect TM4SF20. *E*, SV589 cells with or without pretreatment with 50 μM FB1 for 2 h were analyzed as described in (*A*). *F*, the amounts of sphingolipids in SV589 cells treated with or without 50 μM FB1 for 2 h followed by cotreatment with 6 μM C_6_-ceramide for 6 h were determined by LC–MS. Results are reported as mean ± SD from triplicate incubation of a typical experiment. ∗*p* < 0.05; ∗∗*p* < 0.01 from two-tailed and unpaired *t* test. *G*, A549/pTM4SF20 cells were treated with 50 μM FB1 for 4 h followed by cotreatment with 6 μM C_6_-ceramide for 16 h as indicated. Cells were harvested for immunoblot analysis with antimyc to detect TM4SF20. FB1, fumonisin B1; pac, photoactivatable and clickable; TM4SF20, transmembrane 4 L6 subfamily member 20; TRAM, translocating chain-associated membrane protein.

To more directly demonstrate the interaction between TRAM proteins and pac-C_7_-Cer, we treated cells with pac-C_7_-Cer and/or C_6_-ceramide, exposed the cells to UV, and performed a click reaction to attach biotin to the clickable ceramide analog. Following precipitation with streptavidin-conjugated beads to pull down proteins attached to biotin, the eluates of the precipitates were subject to SDS-PAGE followed by immunoblot analysis. Both TRAM1 and TRAM2 were detected in the eluates when cells were treated with pac-C_7_-Cer but not C_6_-ceramide (Fig. 2*A*, *upper two panels*, lanes 2 and 3). Importantly, increased production of ceramide caused by cotreatment with excess C_6_-ceramide competed with the clickable compound for this binding, leading to marked reduction in the amounts of both proteins in the eluates (Fig. 2*A*, *upper two panels*, lane 4). In addition to exogenous ceramide, endogenous ceramide produced by treatment with bacterial sphingomyelinase (Figs. 1*A* and 2*B*) also competed with the clickable compound for this interaction (Fig. 2*A*, *upper two panels*, lane 5). In order for the competition to occur, the pac-C_7_-Cer concentration used in this experiment was much less than that used in Figure 1*D*. As a result, no size shift was observed for the TRAM proteins upon their interaction with the compound. In contrast to TRAM proteins, Sec61α was not precipitated in any of these experimental conditions (Fig. 2*A*, the *third panel*).

Results shown previously suggest that TRAM proteins specifically bind to the ceramide analog. However, it remained unclear whether they bound to the artificial sphingolipid with a shorter amide-linked acyl chain or the naturally existing long-chain ceramides converted from the analog. To address this question, we treated cells with *N*-hexanoyl-l-erythro-sphingosine (C_6_-L-ceramide), a stereoisomer of C_6_-ceramide that cannot be converted into long-chain ceramide (18). In contrast to C_6_-ceramide, C_6_-l-ceramide was ineffective in competing with the clickable ceramide analog for the interaction with the TRAM proteins (Fig. 2*C*). Consequently, the stereoisomer of C_6_-ceramide was unable to induce RAT of TM4SF20 (Fig. 2*D*). We also addressed this question with FB1 that inhibits conversion of pac-C_7_-Cer to the long-chain bifunctional ceramide analog (Fig. 1*C*). We observed that cotreatment with FB1 inhibited binding of TRAM1 and TRAM2 to molecules derived from pac-C_7_-Cer (Fig. 2*E*). FB1 also inhibited conversion of C_6_-ceramide to ceramide with amide-linked long-chain saturated fatty acid (C14:0) (Fig. 2*F*). Under this condition, the sphingosine produced from C_6_-ceramide was channeled into sphingosine-1-phosphate (Fig. 2*F*). Accordingly, FB1 inhibited RAT of TM4SF20 induced by C_6_-ceramide (Fig. 2*G*). These results suggest that TRAM proteins may have higher affinity toward long-chain ceramides converted from the short-chain analogs, and this interaction may be critical to induce RAT of TM4SF20.

**Figure 3:**
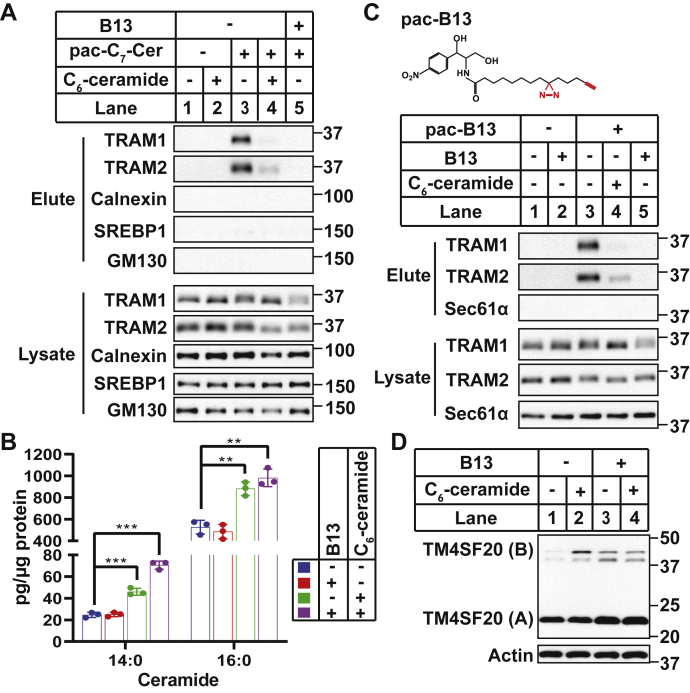
**B13 competes with ceramide to bind TRAM proteins.***A*, SV589 cells treated with or without 10 μM B13 for 1 h were analyzed as described for Figure 2*A*. *B*, SV589 cells treated with 6 μM C_6_-ceramide and/or 10 μM B13 for 6 h were harvested for sphingolipid analysis *via* LC–MS measurement, and the amounts of ceramide with the indicated amide-linked acyl chain are reported as mean ± SD from triplicate incubation of a typical experiment. ∗∗*p* < 0.01; ∗∗∗*p* < 0.001 from two-tailed and unpaired *t* test. *C*, SV589 cells treated with 0.4 μM pac-B13, 80 μM C_6_-ceramide, and/or 10 μM B13 for 1 h were analyzed as described for Figure 2*A*. *D*, A549/pTM4SF20 cells treated with 6 μM C_6_-ceramide and/or 30 μM B13 for 6 h were harvested for immunoblot analysis with antimyc to detect TM4SF20. TM4SF20, transmembrane 4 L6 subfamily member 20; TRAM, translocating chain-associated membrane protein.

We also used B13, a ceramidase inhibitor (19) that should also block conversion of C_6_-ceramide to long-chain ceramides (Fig. 1*A*), to test our hypothesis. As expected, cotreatment with B13 completely inhibited binding of molecules derived from pac-C_7_-Cer with TRAM1 and TRAM2 (Fig. 3*A*). However, lipid measurement results indicated that B13 did not function as a ceramidase inhibitor as predicted under our experimental conditions, as cotreatment with the compound did not prevent C_6_-ceramide from increasing the production of long-chain ceramides (Fig. 3*B*). These results suggest that unlike FB1, B13 does not inhibit binding of molecules derived from pac-C_7_-Cer with TRAM proteins by blocking conversion of the short-chain ceramide analog to long-chain ceramide. Since B13 is structurally similar to ceramide (19), we suspect that the compound might directly bind to TRAM proteins, thereby competitively inhibiting their binding with ceramide. To test this hypothesis, we synthesized a photoactivatable and clickable analog of B13 (Fig. 3*C*). This compound indeed bound to TRAM proteins, and this binding was specific as it was competed by excess unmodified B13 or ceramide derived from exogenous C_6_-ceramide (Fig. 3*C*). In contrast to FB1, treatment with B13 alone induced RAT of TM4SF20 (Fig. 3*D*, lane 3), and cotreatment with C_6_-ceramide did not further enhance this effect (Fig. 3*D*, lane 4). These observations are consistent with the correlation between binding of ceramide analogs to TRAM proteins and RAT of TM4SF20.

**Figure 4:**
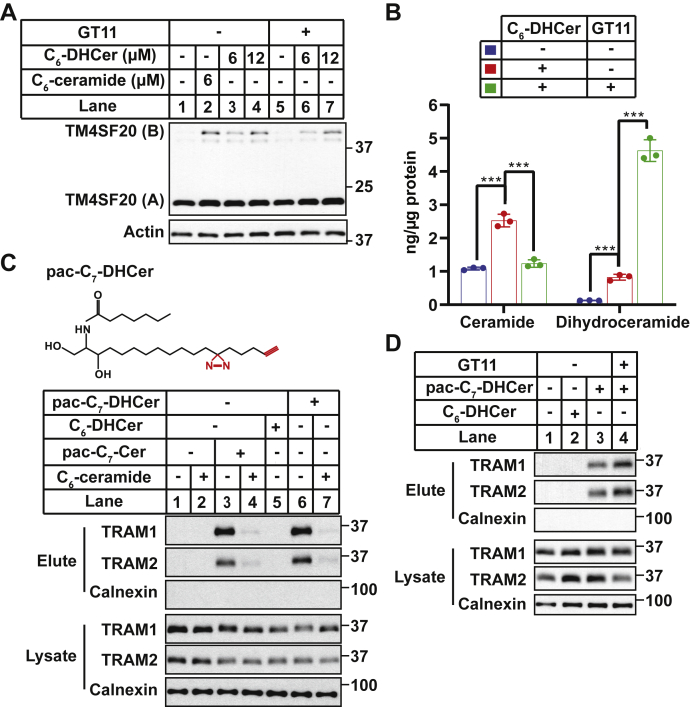
**TRAM proteins interact with dihydroceramide (DHCer).***A*, A549/pTM4SF20 cells treated with indicated amounts of C_6_-ceramide or C_6_-DHCer in the absence or the presence of 1 μM GT11 for 6 h were harvested for immunoblot analysis with antimyc to detect TM4SF20. *B*, SV589 cells treated with C_6_-DHCer and/or GT11 for 6 h were harvested for sphingolipid analysis *via* LC–MS measurement. The amounts of ceramide and DHCer (summation of all species) are reported as mean ± SD from triplicate incubation of a typical experiment. ∗∗∗*p* < 0.001 from two-tailed and unpaired *t* test. *C* and *D*, SV589 cells treated with 0.4 μM pac-C_7_-Cer, 0.4 μM pac-C_7_-DHCer, 80 μM C_6_-ceramide, or 1 μM GT11 as indicated for 1 h were analyzed as described for Figure 2*A*. pac, photoactivatable and clickable; TM4SF20, transmembrane 4 L6 subfamily member 20; TRAM, translocating chain-associated membrane protein.

We previously reported that DHCer stimulated RAT of CCR5 in macrophages (10). Similar to C_6_-ceramide, treatment with C_6_-DHCer also induced RAT of TM4SF20 (Fig. 4*A*, lanes 3 and 4). These observations suggest that DHCer might also be active in inducing RAT of TM4SF20 by binding to TRAM2. However, C_6_-DHCer treatment increased not only the amount of long-chain DHCer but also that of ceramide (Fig. 4*B*), presumably though a reaction catalyzed by DHCer desaturase that converts DHCer to ceramide (Fig. 1*A*). To rule out the possibility that the effect of C_6_-DHCer observed was caused by increased production of long-chain ceramide, we treated the cells with GT11, an inhibitor of the desaturase (20). While inhibiting the conversion of C_6_-DHCer to long-chain ceramide but not long-chain DHCer (Fig. 4*B*), this treatment had no effect on C_6_-DHCer-induced RAT of TM4SF20 (Fig. 4*A*, lanes 5–7). We thus investigated whether TRAM proteins could also bind to DHCer. For this purpose, we synthesized a photoactivatable and clickable analog of C_7_-DHCer (pac-C_7_-DHCer) (Fig. 4*C*). Using the assay shown in Figure 2*A*, we demonstrated that similar to the ceramide analog, TRAM1 and TRAM2 also bound to the DHCer analog (Fig. 4*C*, *upper two panels*, lane 6), and this binding was competed by excess ceramide produced from exogenous C_6_-ceramide treatments (Fig. 4*C*, *upper two panels*, lane 7). Importantly, interaction of TRAM proteins with the clickable analog of DHCer was not inhibited by GT11, which blocks conversion of DHCer to ceramide (Fig. 4*D*). These results suggest that TRAM proteins also interact with DHCer.

**Figure 5:**
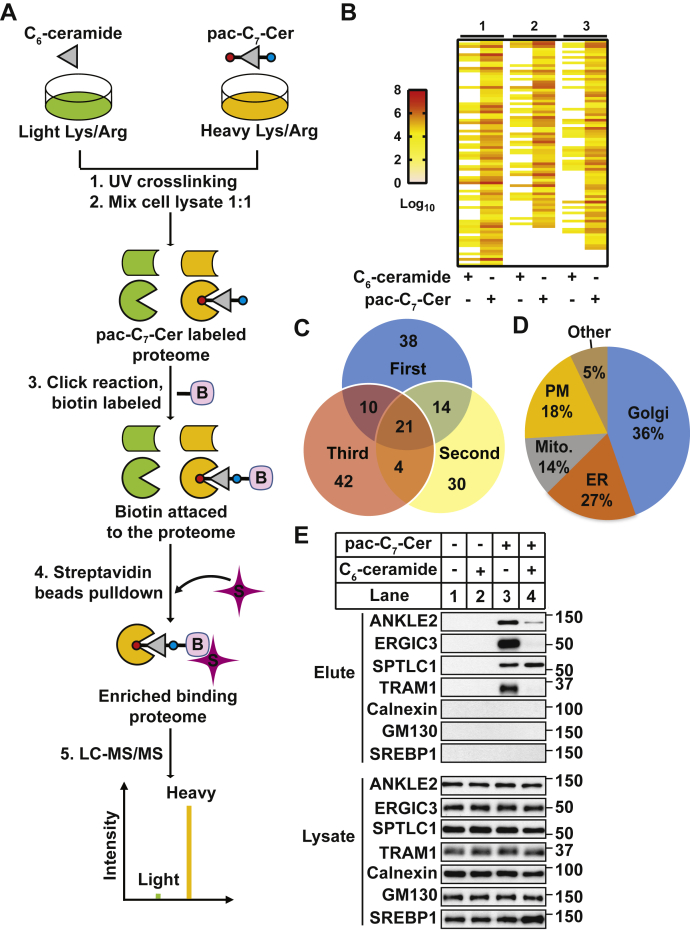
**Identification of ceramide-binding proteins by SILAC-based comparative proteomic analysis.***A*, schematic illustration of experimental design. *B*, a heat map that demonstrates spectral counts of potential ceramide-binding proteins with high confidence in three independent experiments. *C*, overlap quantitation of the potential ceramide-binding proteins in three independent SILAC experiments. *D*, subcellular localization of the potential ceramide-binding proteins identified in three independent SILAC experiments. *E*, SV589 cells treated with 0.4 μM pac-C_7_-Cer, 0.4 μM (lane 2), or 80 μM (lane 4) C_6_-ceramide as indicated for 1 h were analyzed as described for Figure 2*A*. pac, photoactivatable and clickable; SILAC, stable isotope labeling by amino acids in cell culture.

The results shown previously demonstrate pac-C_7_-Cer as a valuable probe to study protein–ceramide interaction. We then investigated whether the compound could be applied to perform proteome-wide identification of ceramide-binding protein through stable isotope labeling by amino acids in cell culture (SILAC)-based MS analysis (Fig. 5*A*). For this purpose, cells cultured in medium containing heavier lysine and arginine were treated with pac-C_7_-Cer. As a control, a parallel batch of the cells cultured in medium containing lighter lysine and arginine was treated with C_6_-ceramide. Following UV exposure, lysates of the cells were mixed at 1:1 ratio and subjected to a click reaction that covalently attached biotin to the clickable compound. Proteins that bound to the clickable compound were isolated by streptavidin-conjugated beads, digested by trypsin, and analyzed by MS. Each peptide should display as a pair of peaks, with the lighter one representing nonspecific binding and the heavier one standing for binding with the clickable ceramide analog. To minimize the nonspecific interaction, we considered proteins with a light to heavy peak ratio (*r*) ≤ 0.1 as those specifically interacting with the ceramide analog. We identified 69 to 77 proteins that met this requirement in each of the three experiments (Fig. 5*B*) (Table S1) and 21 proteins in all three experiments (Fig. 5*C*) (Tables 1 and S1).

**Table 1:** Proteins that potentially bind to the bifunctional ceramide analog

Accession	Protein	Localization	Average ratio of L/H ± SE
A0A087X2B5	BSG	Plasma membrane	0.016 ± 0.005
A0A0A0MR02	VDAC2	Mitochondrion	0.041 ± 0.019
A0A6Q8PFU6	ATP6V0A2	Plasma membrane	0 ± 0
G5E994	GPR107	Plasma membrane	0.010 ± 0.010
H0Y5K5	ERGIC3	Golgi, ER	0.004 ± 0.004
O00461	GOLIM4	Golgi	0.058 ± 0.040
O15269	SPTLC1	ER	0.050 ± 0.028
O43493	TGOLN2	Golgi	0.021 ± 0.021
O43772	SLC25A20	Mitochondrion	0.055 ± 0.043
P07099	EPHX1	ER	0.053 ± 0.008
P21796	VDAC1	Mitochondrion	0.031 ± 0.006
Q02818	NUCB1	Golgi	0.056 ± 0.013
Q10472	GALNT1	Golgi	0.005 ± 0.005
Q15629	TRAM1	ER	0.033 ± 0.033
Q6P1A2	LPCAT3	ER	0.011 ± 0.010
Q86XL3	ANKLE2	ER	0.024 ± 0.005
Q8NBN3	TMEM87A	Golgi	0.000 ± 0.000
Q8NBX0	SCCPDH	Other	0.038 ± 0.038
Q8TB61	SLC35B2	Golgi	0.052 ± 0.012
Q9HC07	TMEM165	Golgi	0.037 ± 0.037
Q9UIQ6	LNPEP	Plasma membrane	0.061 ± 0.031

The majority of the proteins we identified is localized in the Golgi (Fig. 5*D* and Table 1). This observation is consistent with the findings that ceramide is heavily enriched in this organelle (21, 22). Next to the Golgi, ER is the subcellular organelle where most of the potential sphingolipid-interacting proteins were identified by our analysis (Fig. 5*D* and Table 1). These proteins include TRAM1, which has already been demonstrated to bind ceramide in the results shown previously. TRAM2 was identified in one but not all three experiments presumably owing to its lower expression level. Among other ER-resident proteins, we verified that ankyrin repeat and LEM domain–containing protein 2 (ANKLE2), ER–Golgi intermediate compartment protein 3, and serine palmitoyltransferase long-chain base subunit 1 (SPTLC1) bound to pac-C_7_-Cer (Fig. 5*E*, *upper four panels*, lanes 2 and 3) using the assay shown in Figure 2*A*. Excess C_6_-ceramide competed with pac-C_7_-Cer for the interaction with all these proteins except for SPTLC1 (Fig. 5*E*, *upper four panels*, lane 4). In contrast to these proteins, other membrane proteins including GM130, which is located in Golgi where ceramide is heavily enriched, and calnexin, an abundant ER-localized protein, were not precipitated in any of these experimental conditions (Fig. 5*E*, *lower three panels*).

## Discussion

In the current study, we demonstrate that both TRAM proteins bind to ceramide or related sphingolipids. TRAM1 was originally identified as a component of the Sec61 protein translocation channel and was shown to directly interact with nascent peptides during the translocation process (12, 13). While a direct involvement of TRAM2, which is 52% identical to TRAM1, in protein translocation has not been documented, the protein plays a critical role in ceramide-induced RAT of TM4SF20, as knockdown of TRAM2 but not TRAM1 enabled production of TM4SF20(B) with the inverted topology even in the absence of ceramide (7). The finding that TRAM2 binds directly to ceramide suggests that TRAM2 may inhibit the translocation process that produces TM4SF20(B), and this inhibition may be relieved upon its interaction with ceramide. This hypothesis is supported by the correlation between binding of ceramide-related compounds to TRAM2 and their capability to induce RAT of TM4SF20. Another approach to test the hypothesis is to make a mutant TRAM2 defective in ceramide binding and to determine whether expression of this mutant disrupts RAT of TM4SF20. Both TRAM proteins and ceramide synthase contain a TLC domain that was postulated to bind ceramide or related sphingolipids (14). We thus performed alanine scanning of TRAM2 by mutating conserved residues within this domain to alanine. Unfortunately, this approach was inconclusive as the majority of the mutations markedly reduced expression of the protein, presumably affecting proper folding of the protein.

Unlike TRAM2, TRAM1 is not required for RAT of TM4SF20 (7). While the role of TRAM1 in translocation of model peptides during *in vitro* translation was well documented (13, 23), the physiological functions of the protein remain unclear. It will be interesting to determine whether TRAM1 may function similarly to regulate topology of transmembrane proteins different from that regulated by TRAM2 in response to ceramide.

In addition to TRAMs, ceramides signal through various proteins, such as protein phosphatases 1 and 2A (24), protein kinase C zeta (25), and cathepsin D (26). Recent proteomic analyses identified more potential ceramide-binding proteins through biotin-conjugated ceramide analogs (27, 28). A new approach to identify proteins interacting with ceramide is to develop photoactivatable and clickable analogs of sphingolipids. Photoactivatable and clickable analogs of long-chain ceramide (pac-ceramide) were used in some studies to identify ceramide-binding proteins. However, owing to its hydrophobicity, the compound is usually applied *in vitro* to cell homogenates but not used to treat living cells (29, 30), an obstacle that makes it difficult to understand the physiological relevance of the lipid–protein interactions. Other studies employed pac-sphingosine. This compound can be used to treat cells and was successful in identification of some ceramide-binding proteins after the compound was converted to pac-ceramide inside the cells (15, 16). However, pac-sphingosine is not suitable to identify ceramide-binding proteins involved in RAT, as treatment with sphingosine did not induce RAT of TM4SF20. In the current study, we developed pac-C_7_-Cer, a photoactivatable and clickable analog of short-chain ceramide that is active in inducing RAT of TM4SF20. We demonstrated that both TRAM proteins bound to molecules derived from pac-C_7_-Cer. Using this compound as a probe, we showed that TRAM proteins appeared to have higher affinity toward naturally existing long-chain ceramide/DHCer than the short-chain analog. Considering that conversion of ceramide to most other sphingolipids takes place in post-Golgi compartments (31), the ER-resident TRAM proteins are likely to interact with ceramide/DHCer in the ER but not the more complicated sphingolipids.

Besides serving as a probe to study TRAM–ceramide interaction, pac-C_7_-Cer can also be used to perform proteome-wide identification of ceramide-binding proteins. Some of the proteins identified through pac-C_7_-Cer, such as basigin and voltage-dependent anion-selective channel protein 2, were identical to those found in previous studies using pac-sphingosine (15) or pac-Cer (30). However, in contrast to pac-sphingosine that primarily interacted with proteins localized in subcellular organelles involved in endocytosis (15), the potential ceramide-binding proteins identified through pac-C_7_-Cer are enriched in Golgi and ER. We verified SPTLC1, a subunit of SPT that catalyzes the rate-limiting step in *de novo* synthesis of ceramide (32), indeed bound to pac-C_7_-Cer or sphingolipids derived from the compound. These observations might help to explain feedback inhibition of ceramide synthesis. It was reported that a direct interaction of ceramide with a complex formed by orosomucoid-like proteins and SPT is critical for ceramide to exert feedback inhibition on its own synthesis, but the exact component of the complex that binds to ceramide remains unclear (33, 34). The interaction between SPTLC1 and the ceramide analog we observed, if specific, suggests that SPTLC1 may be the binding target for ceramide to exert this reaction. Another ER-localized ceramide-binding protein we verified is ANKLE2. This binding might play an important role in the development of microcephaly, as inactivation of ANKLE2 and loss of *SMPD4*, which encodes a neutral sphingomyelinase that produces ceramide in the ER, all leads to development of the disease (35, 36, 37). Taken together, our study establishes pac-C_7_-Cer as a new tool complementing the existing pac-sphingolipids to investigate ceramide–protein interactions.

## Experimental procedures

### Materials

*N*-hexanoyl-d-erythro-sphingosine (C_6_-ceramide), sphingomyelinase, and [acetonitrile]_4_ CuBF4 were obtained from Sigma. C_6_-l-erythro ceramide, C_6_-DHCer, B13 (D-NMAPPD), and FB1 were obtained from Cayman. Sphingosine was obtained from TOKYO Chemical Industry. GT11 was obtained from Avanti Polar Lipids. Biotin azide was obtained from Click Chemistry Tools. Streptavidin agarose was obtained from TriLink Biotechnologies. ^12^C_6_^14^N_2_-l-lysine, ^12^C_6_^14^N_4_-l-arginine, ^2^H_4_-l-lysine, ^13^C_6_-l-arginine, ^13^C_6_^15^N_2_-l-lysine, ^13^C_6_^15^N_4_-l-arginine, and streptavidin–horseradish peroxidase were obtained from Thermo Fisher Scientific. 3-Azido-7-hydroxycoumarin was obtained from TGI chemicals.

### Synthesis of pac-C_7_-Cer, pac-C_7_-DHCer, pac-B13, and C_7_-ceramide

**Figure 6:**
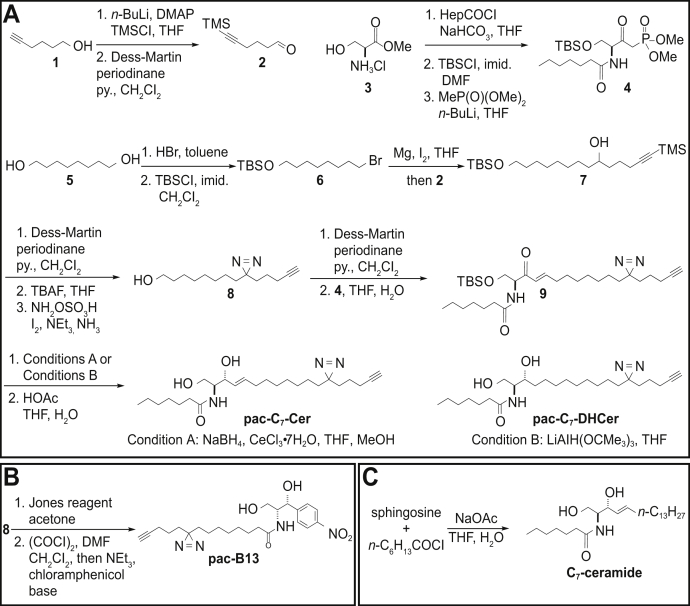
**Synthetic route for pac-C**_**7**_**-Cer, pac-C**_**7**_**-DHCer, pac-B13, and C**_**7**_**-ceramide.***A*, synthesis of pac-C_7_-Cer and pac-C_7_-DHCer hinged on a Horner–Wadsworth–Emmons reaction and chemoselective reduction. *B*, the same photoactivatable and clickable moiety was used for the synthesis of pac-B13. *C*, C_7_-ceramide was prepared from sphingosine and heptanoyl chloride. DHCer, dihydroceramide; pac, photoactivatable and clickable.

Pac-C_7_-Cer was prepared from 3-(8-oxootyl)-3-(4-pentyn-1-yl)-3*H*-diazirin and dimethyl (*S*)-3-heptanamido-4-(*tert*-butyldimethylsiloxy)-2-oxobutylphosphonate through a Horner–Wadsworth–Emmons reaction followed by Luche reduction and silyl deprotection (Fig. 6*A*, condition A). Pac-C_7_-DHCer was prepared similarly with lithium tri-*tert*-butoxyaluminum hydride reduction (Fig. 6*A*, condition B). Pac-B13 was prepared from 3-(7-carboxyheptyl)-3-(4-pentyn-1-yl)-3H-diazirin and chloramphenicol base by amidation (Fig. 6*B*). C_7_-ceramide was prepared by acylation of sphingosine with heptanoyl chloride (Fig. 6*C*).

#### 6-(Trimethylsilyl)hex-5-ynal **(2)**

To a solution of hex-5-yn-1-ol **(1)** (2.2 g, 22.7 mmol, 2.5 ml) and 4-dimethylaminopyridine (560 mg, 4.58 mmol) in tetrahydrofuran (10 ml) was added *n*-butyllithium (1.6 M in hexanes, 50.4 mmol, 31.5 ml) at ‒78 °C slowly. After stirring for 1 h, trimethylsilyl chloride (8.96 g, 82.4 mmol, 10.4 ml) was added, and the solution was stirred at 23 °C for 2 h before quenched with aqueous HCl (1 M and 25 ml) and extracted with ethyl acetate. The combined organic layers were washed with brine, dried over sodium sulfate, concentrated, and purified by silica gel flash column chromatography to give 6-(trimethylsilyl)hex-5-ynol (3.64 g, 94% yield) as a colorless oil. ^1^H NMR (400 MHz, CDCl_3_): δ 3.66 (t, *J* = 6.2 Hz, 2H), 2.26 (*t*, *J* = 6.8 Hz, 2H), 1.77‒1.52 (m, 4H), 0.13 (s, 9H); MS (electrospray ionization [ESI]) calculated for C_9_H_19_OSi (M + H)^+^ 171.1 was found to be 171.1.

To a solution of 6-(trimethylsilyl)hex-5-ynol (2.0 g, 11.74 mmol) and pyridine (3.1 g, 38.8 mmol, and 3.1 ml) in methylene chloride (40 ml) was added Dess–Martin periodinane (5.48 g, 12.92 mmol) at 0 °C. After stirring for 1 h, the reaction was quenched with saturated ammonium chloride and extracted with methylene chloride. The combined organic layers were washed with brine, dried over sodium sulfate, concentrated, and purified by silica gel flash column chromatography to give **(2)** (1.8 g, 90% yield) as a colorless oil. ^1^H NMR (400 MHz, CDCl_3_) δ 9.81 (s, 1H), 2.59 (t, *J* = 7.2 Hz, 2H), 2.31 (q, *J* = 6.8 Hz, 2H), 1.85 (q, *J* = 7.3 Hz, 2H), 0.14 (s, 9H); MS (ESI) calculated for C_9_H_17_OSi (M + H)^+^ 169.1 was found to be 169.1 (Fig. S1*A*).

#### Dimethyl (*S*)-3-heptanamido-4-(*tert*-butyldimethylsiloxy)-2-oxobutylphosphonate **(4)**

To a solution of sodium bicarbonate (2.7 g, 32.1 mmol) in water (68 ml) was added l-serine methyl ester hydrochloride (2.5 g, 16 mmol) followed by ethyl acetate (68 ml) and heptanoyl chloride (3.0 g, 20 mmol, 3.1 ml) at 23 °C. After stirring vigorously for 1 h, the reaction was quenched with saturated ammonium chloride and extracted with ethyl acetate. The combined organic layers were washed with brine, dried over sodium sulfate, concentrated, and purified by silica gel flash column chromatography to give methyl heptanoyl-l-serinate (2.3 g, 90% yield) as a white solid. ^1^H NMR (400 MHz, CDCl_3_) δ 6.44 (d, *J* = 7.3 Hz, 1H), 4.68 (dt, *J* = 7.4, 3.7 Hz, 1H), 3.95 (m, 2H), 3.79 (s, 3H), 2.74 (t, *J* = 5.8 Hz, 1H), 2.26 (t, *J* = 7.6 Hz, 2H), 1.63 (m, 2H), 1.31 (m, 6H), and 0.87 (t, *J* = 6.4 Hz, 3H); MS (ESI) calculated for C_11_H_22_NO_4_ (M + H)^+^ 232.2 was found to be 232.3.

To a solution of methyl heptanoyl-l-serinate (2.0 g, 8.65 mmol), dimethylformamide (20 ml) was added to imidazole (648 mg, 9.5 mmol) followed by *tert*-butyldimethylsilyl chloride (1.96 g, 13 mmol) at 23 °C. After stirring for 10 h, the reaction was quenched with saturated ammonium chloride and extracted with ethyl acetate. The combined organic layers were washed with brine, dried over sodium sulfate, concentrated, and purified by silica gel flash column chromatography to give methyl *O*-(*tert*-butyldimethylsilyl)-*N*-heptanoyl-l-serinate (1.0 g, 60% yield) as a white solid.^1^H NMR (400 MHz, CDCl_3_) δ 6.26 (d, *J* = 7.8 Hz, 1H), 4.67 (dt, *J* = 8.2, 2.8 Hz, 1H), 4.04 (dd, *J* = 10.1, 2.7 Hz, 1H), 3.80 (dd, *J* = 10.1, 3.1 Hz, 1H), 3.73 (s, 3H), 2.24 (dd, *J* = 8.2, 6.9 Hz, 2H), 1.63 (p, *J* = 7.6 Hz, 2H), 1.35 to 1.24 (m, 6H), 0.85 (m, 12H), 0.01 (s, 3H), 0.00 (s, 3H); MS (ESI) calculated for C_17_H_36_NO_4_Si (M + H)^+^ 346.2 was found to be 346.3.

To a solution of dimethyl methylphosphonate (0.69 g, 5.53 mmol, 0.6 ml) was added *n*-butyllithium (2.5 M in tetrahydrofuran, 5.53 mmol, 2.2 ml) at −78 °C. After stirring for 30 min, methyl *O*-(*tert*-butyldimethylsilyl)-*N*-heptanoyl-l-serinate (0.5 g, 1.84 mmol) was added, and the solution was stirred at −78 °C for 2 h and 0 °C for 1 h before quenched with saturated ammonium chloride and extracted with ethyl acetate. The combined organic layers were washed with brine, dried over sodium sulfate, concentrated, and purified by silica gel flash column chromatography to give **(4)** (790 mg, 90% yield) as a white solid. ^1^H NMR (400 MHz, CDCl_3_) δ 6.61 (d, *J* = 7.3 Hz, 1H), 4.72 (dt, *J* = 7.7, 3.9 Hz, 1H), 4.08 (dd, *J* = 10.5, 3.6 Hz, 1H), 3.76 (d, *J* = 3.7 Hz, 3H) 3.73 (d, *J* = 3.5 Hz, 3H), 3.38 (dd, *J* = 22.3, 14.3 Hz, 1H), 3.07 (dd, *J* = 22.5, 14.3 Hz, 1H), 2.21 (p, *J* = 7.7 Hz, 2H), 1.60 (p, *J* = 7.7 Hz, 2H), 1.33 to 1.19 (m, 6H), 0.82 (m, 12H), 0.00 (d, *J* = 3.1 Hz, 6H); MS (ESI) calculated for C_19_H_41_NO_6_PSi (M + H)^+^ 438.2 was found to be 438.3 (Fig. S1*B*).

#### 8-(*Tert*-butyldimethylsiloxy)octyl bromide **(6)**

To a solution of 1,8-octanediol (5.0 g, 34.2 mmol) in toluene (80 ml) was added hydrogen bromide (48% in water, 42.7 mmol, 4.84 ml). After stirring under reflux for 8 h with a Dean–Stark apparatus, the mixture was cooled to room temperature, washed twice with water and once with brine, dried over sodium sulfate, and concentrated to give crude 8-bromooctan-1-ol as a colorless oil. ^1^H NMR (400 MHz, CDCl_3_) δ 3.61 (t, *J* = 6.6 Hz, 2H), 3.39 (t, *J* = 6.8 Hz, 2H), 1.83 (p, *J* = 7.0 Hz, 2H), 1.69 (s, 1H), 1.54 (p, *J* = 6.7 Hz, 2H), 1.47 to 1.35 (m, 2H), 1.36 to 1.27 (m, 6H); MS (ESI) calculated for C_8_H_18_BrO (M + H)^+^ 209.1 was found to be 209.1.

To a solution of crude 8-bromooctan-1-ol obtained previously in methylene chloride (70 ml) at 0 °C was added imidazole (2.55 g, 37.4 mmol). After stirring for 15 min, *tert*-butyldimethylsilyl chloride (7.7 g, 51 mmol) was added, and the solution was stirred at 23 °C for 1 h before quenched with saturated ammonium chloride and extracted with methylene chloride. The combined organic layers were washed with brine, dried over sodium sulfate, concentrated, and purified by silica gel flash column chromatography to give **(6)** quantitatively as a colorless oil. ^1^H NMR (400 MHz, CDCl_3_) δ 3.60 (t, *J* = 6.6 Hz, 2H), 3.40 (t, *J* = 6.9 Hz, 2H), 1.99 to 1.77 (m, 2H), 1.61 to 1.24 (m, 10H), 0.90 (s, 9H), 0.06 (s, 6H); MS (ESI) calculated for C_14_H_32_BrOSi (M + H)^+^ 323.1 was found to be 323.2 (Fig. S1*C*).

#### 14-(*Tert*-butyldimethylsiloxy)-1-(trimethylsilyl)tetradec-1-yn-6-ol **(7)**

To a mixture of magnesium chips (26.2 mg, 1.08 mmol) and iodine (trace) in tetrahydrofuran was added **(6)** (348 mg, 1.08 mmol) under reflux. After stirring for 2.5 h, the mixture was cooled to 0 °C, and a solution of **(2)** (121 mg, 0.72 mmol) in tetrahydrofuran (0.5 ml) was added. After stirring at 23 °C for 1 h, the reaction was quenched with saturated ammonium chloride and extracted with methylene chloride. The combined organic layers were washed with brine, dried over sodium sulfate, concentrated, and purified by silica gel flash column chromatography to give **(7)** (208 mg, 70% yield) as a colorless oil. ^1^H NMR (400 MHz, CDCl_3_) δ 3.59 (m, 1H), 3.58 (t, *J* = 6.6 Hz, 2H), 2.23 (t, *J* = 6.4 Hz, 2H), 1.71 to 1.37 (m, 18H), 0.87 (s, 9H), 0.13 (s, 9H), 0.03 (s, 6H); MS (ESI) calculated for C_23_H_49_O_2_Si_2_ (M + H)^+^ 413.3 was found to be 413.3 (Fig. S1*D*).

#### 3-(8-Hydroxyoctyl)-3-(4-pentyn-1-yl)-3*H*-diazirin **(8)**

To a solution of **(7)** (88 mg, 0.213 mmol) in methylene chloride (2 ml) was added pyridine (50 mg, 0.639 mmol, 50 μl) followed by Dess–Martin periodinane (99 mg, 0.234 mmol) at 0 °C. After stirring for 1 h, the reaction was quenched with saturated ammonium chloride and extracted with ether. The combined organic layers were washed with brine, dried over sodium sulfate, concentrated, and purified by silica gel flash column chromatography to give 14-(*tert*-butyldimethylsiloxy)-6-oxo-1-(trimethylsilyl)tetradec-1-yne (70 mg, 80% yield) as a colorless oil. ^1^H NMR (400 MHz, CDCl_3_) δ 3.58 (t, *J* = 6.6 Hz, 2H), 2.52 (t, *J* = 7.3 Hz, 2H), 2.39 (t, *J* = 7.5 Hz, 2H), 2.25 (t, *J* = 6.9 Hz, 2H), 1.85 to 1.40 (m, 6H), 1.27 (m, 8H), 0.88 (s, 9H), 0.14 (s, 9H), 0.04 (s, 6H); MS (ESI) calculated for C_23_H_47_O_2_Si_2_ (M + H)^+^ 411.3 was found to be 411.4.

To a solution of 14-(*tert*-butyldimethylsiloxy)-6-oxo-1-(trimethylsilyl)tetradec-1-yne (70 mg, 0.17 mmol) in tetrahydrofuran (2 ml) was added tetra-*n*-butylammonium fluoride (1 M in tetrahydrofuran, 0.51 mmol, 0.51 ml) at 0 °C. After stirring overnight at 23 °C, the reaction was quenched with saturated ammonium chloride and extracted with ethyl acetate. The combined organic layers were washed with brine, dried over sodium sulfate, concentrated, and purified by silica gel flash column chromatography to give 6-oxotetradec-1-yn-14-ol (35.6 mg, 93% yield) as a colorless oil. ^1^H NMR (400 MHz, CDCl_3_) δ 3.61 (t, *J* = 6.6 Hz, 2H), 2.53 (t, *J* = 7.2 Hz, 2H), 2.39 (t, *J* = 7.4 Hz, 2H), 2.21 (t, *J* = 6.9 Hz, 2H), 1.95 (t, *J* = 2.7 Hz, 1H), 1.77 (p, *J* = 7.1 Hz, 2H), 1.61 to 1.48 (m, 5H), 1.40 to 1.20 (m, 8H); MS (ESI) calculated for C_14_H_25_O_2_ (M + H)^+^ 225.2 was found to be 225.2.

6-Oxotetradec-1-yn-14-ol (250 mg, 1.1 mmol) was dissolved in ammonia in methanol (7 N in methanol, 15 ml) and stirred at −78 °C for 3 h. Hydroxylamine-*O*-sulfonic acid (189 mg, 1.67 mmol) was then added, and the mixture was warmed to 23 °C. After stirring overnight, ammonia was removed by blowing air through the mixture. The suspension was then filtered, and the filtrate was concentrated. The resulting residue was redissolved in MeOH (0.2 ml) and triethylamine (0.6 ml), and iodine was added at 0 °C until the purple color persists. After stirring at 23 °C for 2 h, the reaction was quenched with saturated ammonium chloride and extracted with ethyl acetate. The combined organic layers were washed with brine, dried over sodium sulfate, concentrated, and purified by silica gel flash column chromatography to give **(8)** (84 mg, 32% yield) as a colorless oil (Fig. S1*E*).

#### (*S*,*E*)-3-(11-heptanamido-12-(*tert*-butyldimethylsiloxy)-10-oxododec-8-enyl)-3-(4-pentyn-1-yl)-3*H*-diazirin **(9)**

To a solution of **(8)** (84 mg, 0.355 mmol) in methylene chloride (3 ml) was added Dess–Martin periodinane (166 mg, 0.39 mmol) at 0 °C. After stirring for 1 h, the reaction was quenched with saturated sodium bicarbonate and extracted with ethyl acetate. The combined organic layers were washed with brine, dried over sodium sulfate, concentrated, and purified by silica gel flash column chromatography to give 3-(8-oxooctyl)-3-(4-pentyn-1-yl)-3*H*-diazirin (66 mg, 79% yield) as a colorless oil. ^1^H NMR (400 MHz, CDCl_3_) δ 9.76 (s, 1H), 2.42 (t, *J* = 7.4 Hz, 2H), 2.16 (t, *J* = 6.9 Hz, 2H), 1.95 (t, *J* = 2.7 Hz, 1H), 1.64 to 1.56 (m, 2H), 1.53 to 1.46 (m, 2H), 1.40 to 1.19 (m, 10H), 1.14 to 1.02 (m, 2H); MS (ESI) calculated for C_14_H_23_N_2_O (M + H)^+^ 235.2 was found to be 235.2.

To a solution of 3-(8-oxooctyl)-3-(4-pentyn-1-yl)-3*H*-diazirin (123 mg, 0.282 mmol) in tetrahydrofuran (1.5 ml) and water (2 ml) was added potassium carbonate (52 mg, 0.376 mmol) at 23 °C. After stirring for 5 min, **(4)** (44 mg, 0.188 mmol) in tetrahydrofuran (0.5 ml) was added. After stirring at 45 °C overnight, the reaction was quenched with saturated ammonium chloride and extracted with ethyl acetate. The combined organic layers were washed with brine, dried over sodium sulfate, concentrated, and purified by silica gel flash column chromatography to give **(9)** (64.8 mg, 63% yield) as a white solid. ^1^H NMR (400 MHz, CDCl_3_) δ 6.97 (dt, *J* = 15.7, 6.9 Hz, 1H), 6.49 (d, *J* = 7.3 Hz, 1H), 6.25 (m, 1H), 4.86 (ddd, *J* = 7.5, 4.5, 3.2 Hz, 1H), 3.99 (dd, *J* = 10.2, 3.2 Hz, 1H), 3.83 (dd, *J* = 10.2, 4.5 Hz, 1H), 2.27 to 2.19 (m, 4H), 2.15 (td, *J* = 7.0, 2.6 Hz, 2H), 1.94 (t, *J* = 2.6 Hz, 1H), 1.67 to 1.58 (m, 2H), 1.53 to 1.15 (m, 20H), 1.13 to 1.01 (m, 2H), 0.86 (t, *J* = 6.5 Hz, 3H), 0.82 (s, 9H), −0.02 (s, 6H); MS (ESI) calculated for C_31_H_56_N_3_O_3_Si (M + H)^+^ 546.4 was found to be 546.4 (Fig. S1*F*).

#### Pac-C_7_-cer

To a solution of **(9)** (8.7 mg, 0.016 mmol) in tetrahydrofuran (0.2 ml) and methanol (0.5 ml) was added cerium(III) chloride (6.52 mg, 0.0175 mmol) in methanol (0.25 ml) at −30 °C. After stirring for 15 min, a freshly prepared solution of sodium borohydride (0.66 mg, 0.0175 mmol) in methanol (0.25 ml) was added at −30 °C. After stirring for 5 min, the reaction was quenched with saturated ammonium chloride and extracted with ethyl acetate. The combined organic layers were washed with brine, dried over sodium sulfate, and concentrated to give crude (*E*)-(11*S*)-(11-heptanamido-12-(*tert*-butyldimethylsiloxy)-10-hydroxydodec-8-enyl)-3-(4-pentyn-1-yl)-3*H*-diazirin (7.8 mg) as white solid.

To a solution of the crude (*E*)-(11*S*)-(11-heptanamido-12-(*tert*-butyldimethylsiloxy)-10-hydroxydodec-8-enyl)-3-(4-pentyn-1-yl)-3*H*-diazirin obtained previously (7.8 mg, 0.0142 mmol) in tetrahydrofuran (0.2 ml) was added water (0.2 ml) and acetic acid (0.6 ml). After stirring 45 °C for 3 h, the reaction was quenched with saturated ammonium chloride and extracted with ethyl acetate. The combined organic layers were washed with brine, dried over sodium sulfate, concentrated, and purified by HPLC to give pac-C7-Cer (4.5 mg, 66% yield) as a white solid. ^1^H NMR (400 MHz, CDCl_3_) δ 6.28 (s, 1H), 5.77 (dt, *J* = 14.2, 6.8 Hz, 1H), 5.53 (dd, *J* = 15.4, 5.6 Hz, 1H), 4.33 (s, 1H), 3.95 (m, 2H), 3.72 (s, 1H), 2.24 (t, *J* = 7.6 Hz, 2H), 2.16 (td, *J* = 6.9, 2.6 Hz, 2H), 2.05 (q, *J* = 7.2 Hz, 2H), 1.95 (t, *J* = 2.6 Hz, 1H), 1.64 (p, *J* = 7.3 Hz, 2H), 1.49 (dd, *J* = 9.3, 6.6 Hz, 2H), 1.39 to 1.19 (m, 20H), 1.07 (p, *J* = 7.6 Hz, 2H), 0.88 (t, *J* = 6.7 Hz, 3H); MS (ESI) calculated for C_25_H_44_N_3_O_3_ (M + H)^+^ 434.3 was found to be 434.4 (Fig. S2*A*).

#### Pac-C_7_-DHCer

To a solution of **(9)** (7.2 mg, 0.0132 mmol) in tetrahydrofuran (0.5 ml) was added lithium tri-*tert*-butoxyaluminum hydride (1.0 M in tetrahydrofuran, 0.033 mmol, 33 μl) at −78 °C. After stirring for 30 min, the reaction was quenched with saturated ammonium chloride and extracted with ethyl acetate. The combined organic layers were washed with brine, dried over sodium sulfate, and concentrated to give crude (11*S*)-(11-heptanamido-12-(*tert*-butyldimethylsiloxy)-10-hydroxydodecyl)-3-(4-pentyn-1-yl)-3*H*-diazirin as a 2:1 mixture of diastereomers.

To a solution of the crude (11*S*)-(11-heptanamido-12-(*tert*-butyldimethylsiloxy)-10-hydroxydodecyl)-3-(4-pentyn-1-yl)-3*H*-diazirin obtained previously, to tetrahydrofuran (0.25 ml) was added water (0.25 ml) and acetic acid (0.8 ml). After stirring at 45 °C for 3 h, the reaction was quenched with saturated ammonium chloride and extracted with ethyl acetate. The combined organic layers were washed with brine, dried over sodium sulfate, concentrated, and purified by HPLC to give pac-C7-DHCer (1.3 mg, 22% yield) as a white solid. ^1^H NMR (400 MHz, CDCl_3_) δ 6.40/6.19 (s, 1H), 4.16 to 3.65 (m, 4H), 2.30 to 2.24 (m, 2H), 2.19 (m, 2H), 2.04 to 1.72 (m, 7H), 1.67 (p, *J* = 7.3 Hz, 2H), 1.51 (dd, *J* = 9.6, 6.3H, 2H), 1.42 to 1.21 (m, 20H), 1.11 (q, *J* = 7.4 Hz, 2H), 0.91 (t, *J* = 6.4 Hz, 3H); MS (ESI) calculated for C_25_H_46_N_3_O_3_ (M + H)^+^ 436.4 was found to be 436.4 (Fig. S2*B*).

#### Pac-B13

To a solution of **(8)** (16 mg, 0.068 mmol) in acetone (0.5 ml) was added a solution of freshly prepared Jones reagent (2.0 M in acetone, 0.271 mmol, 0.14 ml) at 0 °C. After stirring at 23 °C for 2 h, the reaction was quenched with isopropanol. The mixture was then filtered through Celite, and the filter cake was washed with acetone. The filtrate was dried over sodium sulfate and concentrated to give crude 3-(7-carboxyheptyl)-3-(4-pentyn-1-yl)-3*H*-diazirin (3.7 mg). The crude acid (3.7 mg, 0.0148 mmol) was then dissolved in methylene chloride (0.5 ml) and oxalyl chloride (5.6 mg, 0.044 mmol, 4 μl), and *N*,*N*-dimethylformamide (one drop) was added. After stirring at 23 °C for 2 h, the mixture was concentrated to give crude 3-(7-chlorocarbonylheptyl)-3-(4-pentyn-1-yl)-3*H*-diazirin.

To a solution of chloramphenicol base (4 mg, 0.019 mmol) in methylene chloride (0.5 ml) was added triethylamine (4.5 mg, 0.044 mmol, 6.5 μl) followed by the crude 3-(7-chlorocarbonylheptyl)-3-(4-pentyn-1-yl)-3*H*-diazirin obtained previously at 0 °C. After stirring at 23 °C overnight, the reaction was quenched with sodium bicarbonate, and the mixture was extracted with methylene chloride. The combined organic layers were washed with brine, dried over sodium sulfate, concentrated, and purified by HPLC to give pac-B13 (2.3 mg, 35% yield) as a white solid. ^1^H NMR (400 MHz, CDCl_3_) δ 8.14 (d, *J* = 8.8 Hz, 2H), 7.49 (d, *J* = 8.8 Hz, 2H), 6.05 (d, *J* = 8.0 Hz, 1H), 5.16 (d, *J* = 3.2 Hz, 1H), 4.09 (m, 1H), 3.83 (d, *J* = 4.2 Hz, 2H), 2.27 (t, *J* = 7.5 Hz, 1H), 2.09 (td, *J* = 6.9, 2.7 Hz, 2H), 2.04 (td, *J* = 7.4, 5.4 Hz, 1H), 1.88 (t, *J* = 2.7 Hz, 1H), 1.55 (p, *J* = 7.5 Hz, 2H), 1.46 to 1.39 (m, 4H), 1.35 to 1.21 (m, 6H), 1.19 (s, 3H), 1.06 to 0.92 (m, 3H); MS (ESI) calculated for C_23_H_33_N_4_O_5_ (M + H)^+^ 445.2 was found to be 445.3 (Fig. S2*C*).

#### C_7_-ceramide

To a solution of d-sphingosine (7.5 mg, 0.025 mmol) in tetrahydrofuran (0.25 ml) was added sodium acetate (50% in water, 0.25 ml) followed by heptanoyl chloride (5.6 mg, 0.0375 mmol, 5.8 μl) in tetrahydrofuran (0.25 ml) slowly at 23 °C. After stirring for 30 min, the mixture was diluted with water and extracted with ethyl acetate. The combined organic layers were washed with brine, dried over sodium sulfate, concentrated, and purified by silica gel flash column chromatography to give C7-ceramide (6.9 mg, 67%yield) as a white solid. ^1^H NMR (600 MHz, CDCl_3_) δ 6.28 (d, *J* = 7.6 Hz, 1H), 5.78 (m, 1H), 5.52 (ddd, *J* = 15.4, 6.5, 1.6 Hz, 1H), 4.31 (dd, *J* = 6.5, 4.0 Hz, 1H), 3.95 (dd, *J* = 11.3, 3.8 Hz, 1H), 3.90 (dq, *J* = 7.5, 3.7 Hz, 1H), 3.70 (dd, *J* = 11.3, 3.4 Hz, 1H), 2.35 (brs, 2H), 2.23 (p, *J* = 7.4 Hz, 2H), 2.05 (q, *J* = 7.2 Hz, 2H), 1.63 (p, *J* = 7.6 Hz, 2H), 1.40 to 1.19 (m, 28H), 0.88 (m, 6H); MS (ESI) calculated for C_25_H_49_NNaO_3_ (M + Na)^+^ 434.4 was found to be 434.4 (Fig. S2*D*).

### Plasmids and transfection

pTK-TM4SF20-myc encodes full-length human TM4SF20 followed by five tandem repeats of the Myc epitope tag under the control of the weak thymidine kinase (TK) promoter. SV589 cells were set up on day 0 at a density of 2 × 10^5^ per well in 6-well plates. On day 1, cells were transfected with 1 μg/well of pTK-TM4SF20-myc or an empty vector by X-treme GENE HP (Roche) according to the manufacturer's instruction.

### Cell culture

SV589 (human male transformed fibroblasts) cells were maintained in Dulbecco's modified Eagle's medium (DMEM) with 1 g/l glucose (Sigma), 100 U/ml penicillin, 100 μg/ml streptomycin sulfate (Corning), and 5% fetal calf serum in monolayer at 37 °C in a 5% CO_2_ incubator. A549/pTM4SF20 cells, a clone of lung carcinoma cells stably transfected with Myc-tagged TM4SF20 (6), were maintained in 1:1 mixture of Ham's F12 medium and DMEM (Corning) containing 100 U/ml penicillin, 100 μg/ml streptomycin sulfate, 5% fetal calf serum, and 700 μg/ml G418 (Gibco) in monolayer at 37 °C in a 8.8% CO_2_ incubator.

### Immunoblot

Following SDS-PAGE, proteins were transferred to nitrocellulose membranes (Bio-Rad) and immunoblotted with indicated antibodies at the following concentration or dilution: Actin (Sigma; A2066; 1:50,000), ANKLE2 (Bethyl Laboratories; 1:1000), calnexin (Enzo Life Sciences; 1:3000), ER–Golgi intermediate compartment protein 3 (Proteintech; 1:1000), GM130 (Proteintech; 1:5000), SPTLC1 (Santa Cruz; 1:1000), anti-SREBP1 (2 μg/ml as reported previously (38)), anti-myc IgG-9E10 (1 μg/ml, produced from hybridoma obtained from the American Type Culture Collection), TRAM1 (0.1 μg/ml, a monoclonal antibody generated in this study by immunizing mice with polypeptides corresponding to amino acid residues 278–291 and 361–374 of TRAM1), and TRAM2 (1 μg/ml, a polyclonal antibody generated in this study by immunizing rabbit with polypeptides corresponding to amino acid residues 342–355 of TRAM2). Bound antibodies were visualized with a peroxidase-conjugated secondary antibody (Jackson ImmunoResearch Laboratories; 1:5000) using the SuperSignal ECL-HRP substrate system (Thermo Fisher Scientific).

### Thin layer chromatography

Separation of pac-sphingolipids by thin layer chromatography was performed exactly as previously described (39) except that the analysis was performed on 12 × 6 cm plates, and images were captured under an LED lamp (10 W 420 nm LED) equipped with a 490 nm excitation filter. pacFA Ceramide, pacFA GlcCer, pacFA GalCer, and PhotoClick Sphingosine (all from Avanti Polar Lipids) served as markers for the analysis.

### UV crosslinking and click chemistry reaction

Cells were irradiated for 15 min on ice without plate lids under 306 nm UV light in a UV Stratalinker 2400 apparatus (Stratagene), washed three times with cold PBS, and lysed in 1% (w/v) SDS dissolved in PBS supplemented with freshly prepared Benzonase Nuclease (Sigma; 1:1000 dilution). Lysate containing 0.03 to 0.1 mg protein was subjected to click chemistry reactions to attach biotin *via* Cu (I)-catalyzed azide–alkyne cycloaddition (CuAAC). Briefly, cell lysate was mixed with 100 μM Tris(benzyltriazolylmethyl)amine (dissolved in 4:1 mixture of dimethyl sulfoxide: *t*-butanol), 1 mM Tris(2-carboxyethyl)phosphine (freshly dissolved in water), 1 mM CuSO_4_ (freshly dissolved in water), and 100 μM biotin azide (dissolved in dimethyl sulfoxide). The mixture was rotated at room temperature for 1 h. The click reaction attaching biotin in lysate containing ∼0.3 mg protein was terminated by incubation of the reaction mixture with four volumes of acetone at −80 °C for 30 min. Following centrifugation, the pellets were washed two times with ice-cold acetone, solubilized in 4% SDS dissolved in PBS, and incubated with streptavidin agarose beads prewashed with PBS containing 0.1% Tween (v/v) and 200 μg/ml bovine serum albumin at room temperature for 30 min. The pelleted beads were washed twice with 0.1% Tween dissolved in PBS and twice with 4% SDS dissolved in PBS. The biotin-labeled proteins were eluted from the beads by 4% SDS dissolved in PBS at 95 °C for 20 min.

### Sphingolipid quantitation

Sphingolipids were measured by Metabolic Phenotyping Core in UT Southwestern Medical Center exactly as previously described (4).

### SILAC

SV589 cells were grown in SILAC DMEM medium (Sigma) containing light lysine (^12^C_6_^14^N_2_-l-lysine) and arginine (^12^C_6_^14^N_4_-l-arginine), medium lysine (^2^H_4_-l-lysine) and arginine (^13^C_6_-l-arginine), or heavy lysine (^13^C_6_^15^N_2_-l-lysine) and arginine (^13^C_6_^15^N_4_-l-arginine) supplemented with 5% dialyzed fetal bovine serum (Thermo Fisher Scientific) and penicillin/streptomycin (Corning) for eight cell-doubling times to achieve complete labeling (99% labeling efficiency). In the first experiment, light and medium Lys/Arg-labeled cells were treated with 0.4 μM C_6_-ceramide and pac-C_7_-Cer, respectively, for 1 h. In the second and third experiments, medium and heavy Lys/Arg-labeled cells were treated with 0.4 μM C_6_-ceramide and pac-C_7_-Cer, respectively, for 1 h. After UV crosslinking as described previously, lysates of the cells subjected to different treatments were mixed at 1:1 ratio according to their protein concentration. Following click reaction attaching biotin, the biotinylated proteins were isolated as described previously and separated across 5 to 10 mm in a precast SDS-PAGE gel (Bio-Rad) stained with InstanBlue (Expedeon). Proteins in the stained area were excised and digested overnight with trypsin (Pierce) followed by reduction and alkylation with DTT and iodoacetamide (Sigma–Aldrich). The samples then underwent solid-phase extraction cleanup with an Oasis MCX plate (Waters), and the resulting samples were injected onto an Orbitrap Fusion Lumos mass spectrometer coupled to an Ultimate 3000 RSLC-Nano liquid chromatography system. Samples were injected onto a 75 μm i.d., 75-cm long EasySpray column (Thermo Fisher Scientific) and eluted with a gradient from 0 to 28% buffer B over 90 min. Buffer A contained 2% (v/v) acetonitrile and 0.1% formic acid in water, and buffer B contained 80% (v/v) acetonitrile, 10% (v/v) trifluoroethanol, and 0.1% formic acid in water. The mass spectrometer operated in positive ion mode with a source voltage of 1.5 kV and an ion transfer tube temperature of 275 °C. MS scans were acquired at 120,000 resolution in the Orbitrap, and up to 10 MS/MS spectra were obtained in the ion trap for each full spectrum acquired using higher-energy collisional dissociation for ions with charges 2 to 7. Dynamic exclusion was set for 25 s after an ion was selected for fragmentation.

Raw MS data files were analyzed using Proteome Discoverer, version 2.4.1.15 (Thermo Fisher Scientific), with peptide identification performed using Sequest HT searching against the human protein database from UniProt (downloaded January 8, 2021, containing 75,552 sequences). Fragment and precursor tolerances of 10 ppm and 0.6 Da were specified, respectively, and three missed cleavages were allowed for a trypsin digestion (cleavage after K and R except when followed by P). Carbamidomethylation of Cys was set as a fixed modification. Oxidation of Met, ^2^H_4_-lysine, ^13^C_6_-arginine, ^13^C_6_^15^N_2_-lysine, and ^13^C_6_^15^N_4_-arginine was set as variable modifications on all peptides. Acetylation, methionine loss, and methionine loss + acetylation were included as variable protein N-terminal modifications. The false discovery rate cutoff was 5% for all peptides. Protein abundances were determined by summing the heights of all identified peptides for that protein. Ratios of protein abundancy between samples obtained from cells cultured in light, medium, or heavy medium were reported in Table 1.

## Data availability

All data are contained within the article. The raw SILAC data were deposited in MassIVE with the URL: ftp://MSV000088110@massive.ucsd.edu.

## Supporting information

This article contains supporting information.

## Conflict of interest

The authors declare that they have no conflicts of interest with the contents of this article.
