# Supplementary material for: Identification of TRAMs as sphingolipid-binding proteins using a photoactivatable and clickable short-chain ceramide analog
Source: J Biol Chem. 2021 Nov 16;297(6):101415. doi: 10.1016/j.jbc.2021.101415 (PMC8665359; doi:10.1016/j.jbc.2021.101415)
Supplement: Figures S1 and S2 [file mmc1.pdf]

Identification of TRAMs as Sphingolipid-Binding Proteins Probed by a Photoactivatable and Clickable Short Chain Ceramide Analog

Yaqin Deng, Lin You, Yong Lu, Sungwon Han, Jingcheng Wang, Nikitha Vicas, Chuo Chen, and Jin Ye

This supporting information contains two supporting figures and one supporting table.

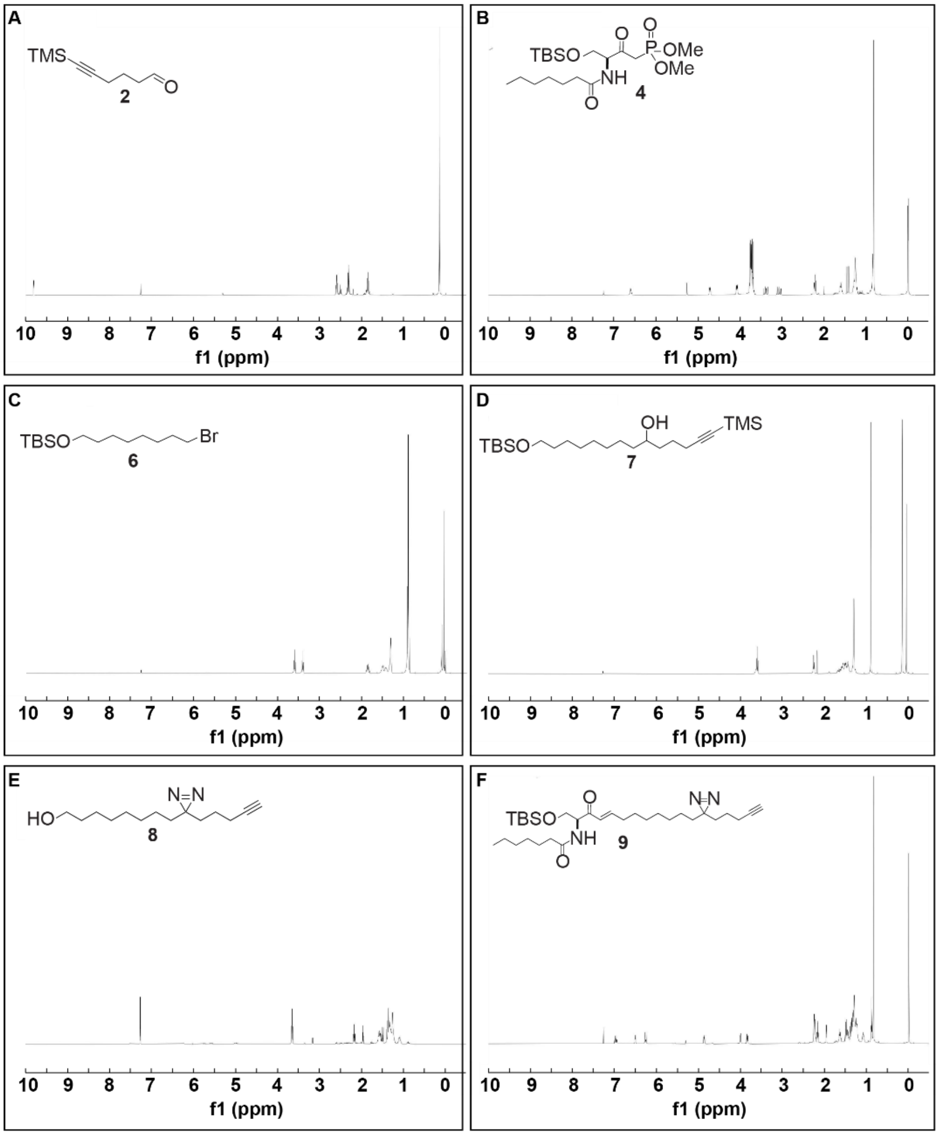

Figure S1. ^{1}H NMR spectrum of intermediate compounds involved in synthesis of pac-C_7-Cer

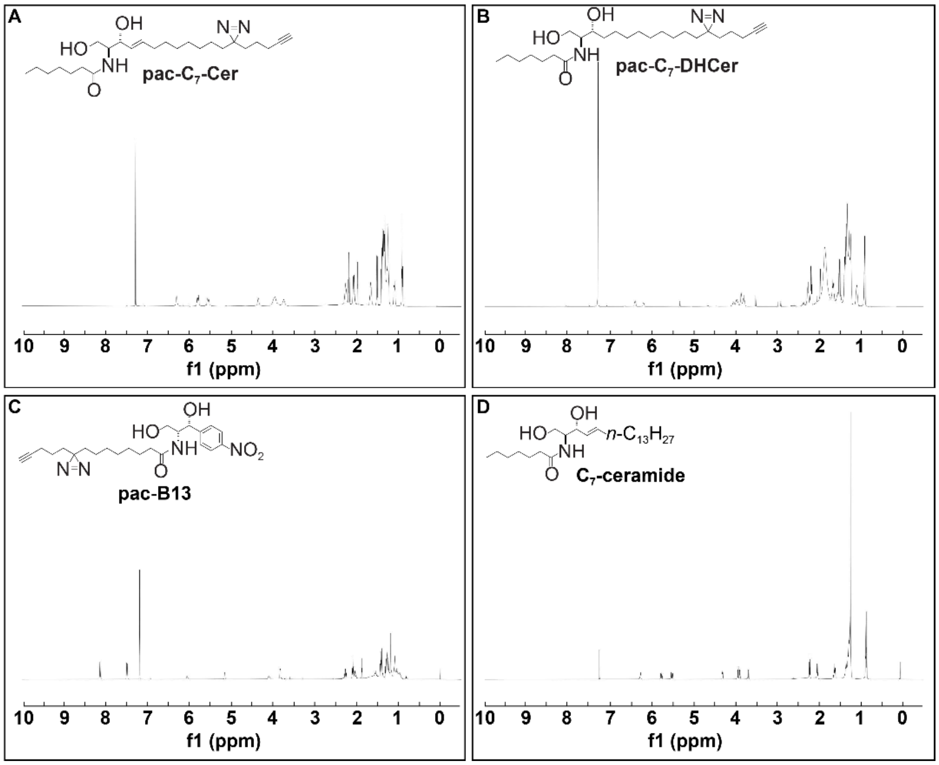

Figure S2. ^{1}H NMR spectrum of the final products.

Table S1. Potential ceramide-binding proteins identified in three independent experiments through SILAC
